# Functional diversity of microglia – how heterogeneous are they to begin with?

**DOI:** 10.3389/fncel.2013.00065

**Published:** 2013-05-14

**Authors:** Uwe-Karsten Hanisch

**Affiliations:** Institute of Neuropathology, University of GöttingenGöttingen, Germany

**Keywords:** diversity, cytokines, immunity, innate, microglia, subtypes, TLR, experience

## Abstract

Microglia serve in the surveillance and maintenance, protection and restoration of the central nervous system (CNS) homeostasis. By their parenchymal location they differ from other CNS-associated myeloid cells, and by origin as well as functional characteristics they are also–at least in part–distinct from extraneural tissue macrophages. Nevertheless, microglia themselves may not comprise a uniform cell type. CNS regions vary by cellular and chemical composition, including white matter (myelin) content, blood–brain barrier properties or prevailing neurotransmitters. Such a micromilieu could instruct as well as require local adaptions of microglial features. Yet even cells within circumscribed populations may reveal some specialization by subtypes, regarding house-keeping duties and functional capacities upon challenges. While diversity of reactive phenotypes has been established still little is known as to whether all activated cells would respond with the same program of induced genes and functions or whether responder subsets have individual contributions. Preferential synthesis of a key cytokine could asign a master control to certain cells among a pool of activated microglia. Critical functions could be sequestered to discrete microglial subtypes in order to avoid interference, such as clearance of endogenous material and presentation of antigens. Indeed, several and especially a number of recent studies provide evidence for the constitutive and reactive heterogeneity of microglia by and within CNS regions. While such a principle of “division of labor” would influence the basic notion of “the” microglia, it could come with the practival value of addressing separate microglia types in experimental and therapeutic manipulations.

## INTRODUCTION

Microglia are myeloid cells of the central nervous system (CNS). They are found with parenchymal distribution throughout the various regions of the brain, the spinal cord and also in the retina where they engage with numerous tasks in the surveillance and maintenance of the tissue homeostasis as well as the protection of the structural and functional integrity of the CNS (Hanisch and Kettenmann, 2007; Ransohoff and Cardona, 2010; Kettenmann et al., 2011; Prinz et al., 2011; Saijo and Glass, 2011). We are currently vitnessing an enormous development in microglial research as literally all aspects of their physiology undergo in-depth clarification and revision. Was it for a long time a common believe that microglia are under healthy conditions “resting” and functionally dormant it is now clear that they actively monitor their local environment with motile processes, nurse synapses in periodical interactions and assist in the myelin turnover by the clearance of oligodenroglia-derived exosomes (Davalos et al., 2005; Nimmerjahn et al., 2005; Haynes et al., 2006; Wake et al., 2009; Graeber, 2010; Fitzner et al., 2011; Paolicelli et al., 2011; Tremblay et al., 2011; Schafer et al., 2012; Kettenmann et al., 2013). Microglia participate in the development and plasticity of the CNS (Saijo and Glass, 2011; Tremblay et al., 2011; Kettenmann et al., 2013). Fundamental questions concerning their developmental origin and fate from embryonic states to aging, their turnover under normal (healthy) and replenishment under disease conditions have been addressed as well, employing sophisticated approaches for unraveling lineage relations and principles of microglial in-tissue maintenance (Mildner et al., 2007; Ginhoux et al., 2010; Ajami et al., 2011; Schulz et al., 2012; Gomez Perdiguero et al., 2013; Greter and Merad, 2013; Kierdorf et al., 2013; Yona et al., 2013). Most notably, features and functions as sentinels and innate immune cell are of foremost interest as they regard crucial roles played by microglia in emergency situations and chronic diseases.

Their striking ability to rapidly react to infection, trauma, ischemia or other real or potential threats has been known as microglial “activation”. However, this term does not adequately reflect the diversity of response options, nor does it define the net impact on the CNS (Hanisch and Kettenmann, 2007; Kettenmann et al., 2011; Hanisch, 2012). Against earlier notions that dominated the text book knowledge for a long time, microglia are not notorious miscreants lurking in the CNS to harm neurons on any occasion. They are not placed there simply as a risk factor. Their association with a lesion does not automatically identify them as a harming component, and even proven involvement in neuropathologic events and processes might be outnumbered by the cases in which their activity limits the damage or eliminates a localized minor defect. However, most of their protective actions probably remain unnoticed, while our view on pathological implications got biased for situations in which such activities failed (Schwartz et al., 2006; Hanisch and Kettenmann, 2007). On the other hand, harmful contributions of microglia have been convincingly demonstrated, indicating that the actual impact can differ dramatically. Indeed, eligibility for diverse responses and complexity of the functional repertoire are key elements in microglial physiology.

The concept of reactive phenotypes, originally and largely founded by investigations on extraneural macrophages, has meanwhile been expanded to microglia (Mantovani et al., 2004; Town et al., 2005; Butovsky et al., 2006c; Schwartz et al., 2006; Hanisch and Kettenmann, 2007; Mosser and Edwards, 2008; Ransohoff and Perry, 2009; David and Kroner, 2011; Olah et al., 2011). It encompasses the recognition of a plethora of “activating” signals, their integration in the context of a given situation and their translation into adapted profiles of induced (or suppressed) genes and functions (Gordon and Taylor, 2005; Mosser and Edwards, 2008; Murray and Wynn, 2011; Hanisch, 2012, 2013). It also allows to better understand at the molecular, cellular and systemic level why and how microglial activation can result in rather protective outcomes or in a worsening of the damage. Proper selection and initiation, maturation and termination of reactive phenotypes are essential not only for the immediate responses to acute insults. They are immanent to the development and progression of autoimmune, neurodegenerative and age-related diseases, primary tumors and metastases, certain neuropsychiatric disorders as well as the compensatory and restorative attempts of the CNS (Miller and Streit, 2007; Perry et al., 2007; Monji et al., 2009; Pollard, 2009; Neumann et al., 2009; Chen et al., 2010; Prinz et al., 2011; Derecki et al., 2012; Cunningham, 2013; Aguzzi et al., 2013).

A question not yet addressed properly concerns the simple reflection as to whether the large number of microglia in a CNS represents a homogeneous population with identical duties and functional capacities. Microglia can account for 5–12% of the cell numbers, depending on the anatomical region (Lawson et al., 1990). These regions differ by biochemical and cellular composition, circuitry and functions – and thus probably differ by their needs of support and assistance. Diversity in morphology, physiology and even aspects of immunity is established or getting more and more recognized for subtypes of neurons and astrocytes (Landgraf and Evers, 2005; Matyash and Kettenmann, 2010; Cho et al., 2013). Immune cells, such as lymphocytes, dendritic and also monocytic cells are classified by subsets that diversify by expression patterns and functions (Yona and Jung, 2010; Satpathy et al., 2012). Microglia, however, the principal immunocompetent element of the CNS, would still be considered as a uniform community with standardized properties, just varying by regional density, obscure cell shape parameters or some protein expression levels? This is rather unlikely. It appears especially doubtful in the light of evidence for constitutive heterogeneity and responder diversity.

**FIGURE 1:**
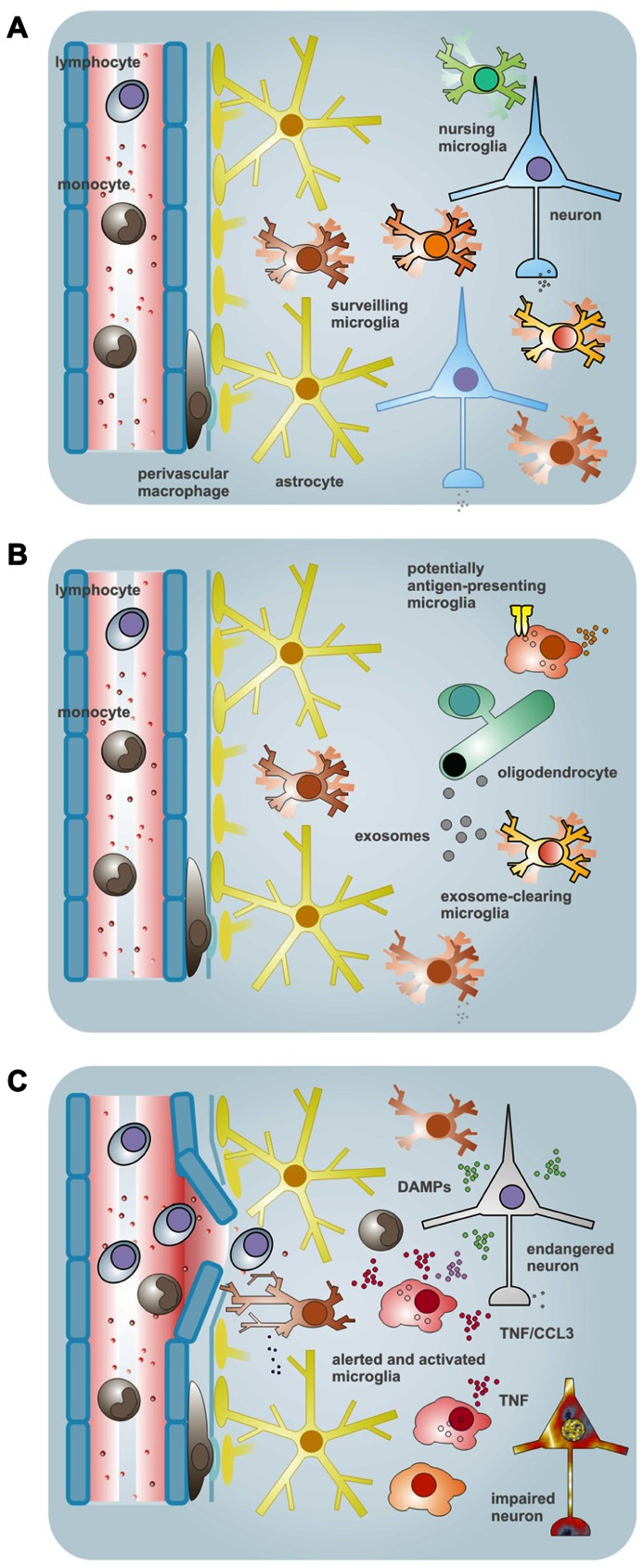
**Schematic overview of the conceptual framework and some supporting findings pointing to a heterogeneity of microglial properties**. **(A)** In the normal CNS tissues, microglia comprise a population of myeloid cells with parenchymal distribution. The ramified morphology and a low expression of immune function-related molecules were formerly taken as signs of a “resting” status. This view got corrected upon demonstration of active tissue surveillance and periodic inspection of synapses with their motile processes. Nevertheless, by morphology and immunophenotype, microglia may still largely appear as a homogeneous cell type, although regional differences exist regarding density, morphology, capacity for proliferation and expression of certain proteins (or antigenic structures). On the other hand, subtle or yet unidentified variations in house-keeping duties (such as the nursing of synapses) and latent capacities may exist that define subtypes among and within anatomical subdivisions. **(B)** One of the duties unequally performed by microglia under physiological conditions relates to the macropinocytotic uptake of myelin-laden exosomes as they are emitted by oligodendrocytes in a principle of “outsourced” myelin turnover. Upon a challenge, such as by IFNγ, this subset of microglia reveals a lack of MHC II expression, in opposition to a largely complementary portion of cells that readily upregulate the surface structure for (potential) professional antigen presentation – but, in turn, do not engage with the exosomal clearance. As a purpose of this division of labor, immunologically silent disposal of endogenous material can take place in a sequestered compartment. **(C)** Challenges by bacterial agents, like LPS, or probably also appearance of endogenous factors with a connotation of damage and a similar TLR4-agonistic activity, can induce the synthesis of TNFα, which can then affect the vitality and functionality of resident as well as infiltrating (immune) cells. Since the production appears to be a privilege of some microglia only (even within local cell communities), they could claim the role of an instructor role or *maître de plaisir* during a response. TNFα-producing cells further subdivide by the ability or inability to also release CCL3. Yet the organization of such responder subtypes could be based on entirely different principles. Cellular subsets could be predetermined as to their functional capacities, acquire such distinction by environmental cues or regulate activities in a stochastic process. The scheme was adapted from previous own work (Hanisch and Kettenmann, 2007; Scheffel et al., 2012).

While this idea still awaits more experimental validation as well as conceptual maturation, first findings and interpretations, implications and consequences have been presented and discussed previously already (Hanisch and Kettenmann, 2007; Hanisch, 2012, 2013), and essentials as well as new elements will be stressed below. They concern a principle by which some microglia can act as a master subset due to a privileged release of a cytokine, such as tumor necrosis factor α (TNFα). Such a cell may take the role of a *primus inter pares* within a local community to govern activities of neighboring cells (**Figure 1**). They also address the preferential expression of major histocompatibility complex (MHC) structures for antigen presentation and of a molecular machinery that would allow a discrete routing of phagocytotic cargo to subsets of microglia. Organized in distinct subpopulations potentially interfering functions could thereby segregate. Notably, the reactive phenotypes with their profiles of transcribed genes and executed functions could build up on the individual contributions of responding sets of microglia, rather than representing the mere sum of equal performances. Nevertheless, whether and how such specialized capacities are pre-determined or randomly assigned to subsets among a population of reactive microglia is not known yet.

## HETEROGENEITY OF TISSUE MACROPHAGES

Macrophages are found in many tissues and organs. They share a core of duties for which they are equipped with an array of constitutively expressed or inducible molecules, such as receptors, enzymes, or cytokines. While macrophage-associated tasks in homeostatic control or responses to damage and infection obviously rely on similar sets of genes and functions the various tissue environments must also determine adaptations of features. Kupffer cells in the liver, alveolar macrophages in the lung, osteoclasts in the bones or the macrophages in the spleen are embedded within characteristic cellular communities and are exposed to particular bio/chemical milieus. Accordingly, gene expression patterns and the organization of functions under normal conditions and in emergency situations likely require adjustment and control to suite the particular needs. Indeed, sophisticated analyses reveal a substantial diversity in the transcriptomes of tissue macrophages (Gautier et al., 2012) as well as in the factors that guide their development (Greter and Merad, 2013).

### MACROPHAGES AS A FAMILY OF MYELOID CELLS

An assessment of the gene expression profiles in resting peritoneal, pulp splenic and lung macrophages as well as CNS microglia revealed a considerable degree of diversity between these populations, more than among populations of dendritic cells (DCs) studied in parallel (Gautier et al., 2012). Several genes distinguished macrophages from DCs. They comprised a signature that was more or less definite depending on the criteria of strict exclusiveness, levels of expression or presence in all or only some of the macrophage types. It included the immunoglobulin Fc receptors FcγRI (CD64) and FcγRIII (CD16), Toll-like receptor (TLR) 4 and its coreceptor CD14, the receptor of granulocyte colony stimulating factor (G-CSF), the MHC class I-related molecule MR1 and some enzymes with metabolic implications or (other) roles in signaling. Molecules commonly serving detection of (tissue) macrophages – besides F4/80 as the prototypical marker-, such as CD11b (αM integrin, complement receptor 3 CR3), CD68 (macrosialin), or CD206 (mannose receptor), did not fulfill the demanding properties of exclusive and/or panpopulational expression by macrophages (Gautier et al., 2012).

### DIVERSITY AMONG THE MACROPHAGES FROM DIFFERENT TISSUES

Macrophage populations also differ themselves by numerous molecules that they can express. TLRs, C-type lectins as well as chemokine and efferocytic receptors were identified as gene families with heterogeneous expression patterns, thus pointing to tissue-specific adjustments in typical innate immune and macrophage effector activities (Gautier et al., 2012). Other studies on the relatedness of microglia to myeloid cells in particular as well as to immune and non-immune cells in general concluded on a strong similarity to the bone marrow-derived macrophages (BMDM), more than to peritoneal macrophages (Saijo and Glass, 2011). In our own work, when comparing the organization and properties of TLR-stimulated responses, we also see more similarities of microglia to BMDM than to peritoneal macrophages, suggesting that relationships are reflected by the reactive behavior as well. Tissue macrophages may thus vary by molecular equipment due to functional requirements as well as the daily or situational exposure to different signals (Davoust et al., 2008; Murray and Wynn, 2011; Hanisch, 2012). While alveolar macrophages are continuously confronted by airborne microbes, their peritoneal counterparts may occasionally sense traces of agents that derive from commensal microbiota. Microglia, on the contrary, would normally never face confrontations with such factors. Their appearance must then be interpreted as a true threat.

Cluster analyses of gene profiles, protein expression or functional activities in myeloid cells might be less affected by the mouse strain or even the species of the laboratory animals, as long as cell types are compared within each category and assuming that such fundamental relationships are conserved. Nevertheless, systematic comparisons of the mouse and human cell repertoire are still lacking to ensure that classifications relying on animals studies are representative. Comparisons of the molecular and functional features are certainly prone to pitfalls when routine preparations of cells involve animals of different ages. Cultures of microglia served in a countless number of studies and delivered invaluable information on their basic properties (Kettenmann et al., 2011). Yet the popular standard *in vitro* setting uses newborn mice and rats, whereas peritoneal macrophages, for example, are more easily isolated from (young) adults. Differences deliberately assigned to the tissue may thus rather be determined by the ontogenetic stage.

Indeed, microglial cells reveal a remarkable maturation of basic features and response options during postnatal development (Scheffel et al., 2012). Moreover, if a property is well established for these cells, then it is the ability to rapidly react to even subtle changes in their environment as brought about by cellular impairments, blood–brain barrier (BBB) disruption and tissue injury (Hanisch and Kettenmann, 2007). Any preparational procedure that would result in a liberation of microglia-active substances and would allow them to exert influences on gene regulation at the time point of sampling may suffer from confounding responses to damage. Even mild cell isolations unavoidably associate with tissue disintegration. This has been stimulating efforts to develop appropriate techniques by wich undesired stimulation of microglia is largely circumvented or excluded from affecting the actual status of the cells at the time point of their isolation (Mertsch et al., 2001; de Haas et al., 2008). As an alternative, isolation and characterization can be separated in time, to allow accommodation of the cells. Such a strategy bears the risk that features acquired by cells – and in particular microglia – in the intact tissue at a given time, location and state are lost upon sampling and passing through cultivation. Surprisingly, however, critical properties can still be preserved. Analyses of molecular and physiological properties of microglia *ex vivo* under basal and stimulated conditions revealed a developmental profile, with gradual and transient adjustments according to the animal age at cell isolation, rather than as a function of time in culture (Scheffel et al., 2012). Apparently, cells can maintain a status (or critical aspects of) relating to the situation *in vivo*.

### MICROGLIA AS A CNS-SPECIFIC POPULATION OF MYELOID CELLS

Microglia comprise myeloid cells with parenchymal distribution. They thereby differ by location from other cells of the mononuclear phagocyte system that are contained within the bony compartment harboring the CNS, such as the perivascular cells or the macrophages of the choroid plexus and the meninges (Kettenmann et al., 2011). The fine processes that can be visualized by antibody and lectin staining for marker molecules or that are revealed in transgenic animals expressing fluorescent proteins under the control of (more or less) microglia-selective promotors (Kettenmann et al., 2011) were commonly taken as an indication of a “resting” state. As meanwhile known better, this ramified morphology is a cytoarchitectural reflection of their surveillance function in the healthy adult tissue (Nimmerjahn et al., 2005). By territorial organization and with a cell body staying faithful to the location, the processes are in constant motion to sample information about the homeostatic situation, to receive inputs about the activity of nearby neurons (and other glia) and to scan the synaptic interconnections. It is probably a fine-anatomical adaptation to the complex tissue organization that enables microglia to reach with their tiny arms into narrow spaces between axons and dendritic trees without disturbing their structure and functionality. Besides a surveillance, it guarantees precision in shaping neuronal connections, as a suggested role of microglia in CNS development and plasticity as well as in response to altered neuronal activity. The phenomenon, termed synaptic stripping or pruning, has been elegantly shown by a number of studies (Wake et al., 2009; Paolicelli et al., 2011; Schafer et al., 2012; Kettenmann et al., 2013).

The delicate CNS architecture also determines that innate immune reactions of microglia are tightly controlled. The proinflammatory response repertoire is essential for fighting infections, but it also contributes – with many facets – to the events following tissue damage, non-infectious injuries as well as subsequent attempts of repair, while carrying enormous potential for aggravating destructive cascades (Bitsch et al., 2000; Butovsky et al., 2006b; Schwartz et al., 2006; Block et al., 2007; Chen and Nunez, 2010). Uncontrolled inflammation and tissue swelling are not tolerated by the CNS. Upon such disturbances and insults, microglia depart from the surveillance mode and activate programs for executive actions that must be kept in magnitude and organized by quality to serve as much in the defense as simultaneously avoiding unnecessary impairment (Hanisch and Kettenmann, 2007; Kettenmann et al., 2011). Along with a functional transition, microglial processes are rapidly reorientated, the ramified morphology resolves, cells turn into a more ameboid shape and even migrate to the site of a lesion. This remarkable change has been taken as a prototypical sign of a microgial response, and it was noted already at the dawn of microglia research (Kettenmann et al., 2011). Nevertheless, whereas morphology changes with and in support of function, and often also with complex spatiotemporal pattern (Morrison and Filosa, 2013), it is not automatically a reliable surrogate of the actual activities (Ilschner and Brandt, 1996). On the other hand, variations in the cellular shape, as seen in the various anatomical divisions, may still point to adjustments to the environment. When considering heterogeneity of microglia, these adjustments should not be ignored.

While the BBB guarantees a special milieu in terms of the ionic, molecular and cellular CNS composition it is not an impenetrable wall, but rather an interface and check point where a controlled exchange of nutrients and metabolites is managed. Also for the so-called “immune privilege” of the CNS, its roles and mechanisms in immune surveillance and the organization of immune reactions are now better understood as to their anatomical fundaments as well as physiological processes (Bechmann et al., 2007; Ransohoff and Engelhardt, 2012). The BBB yet also differs in organizational features throughout the CNS and can change by integrity and functional properties which, in turn, can affect local populations of microglia. In other words, the CNS and its vascular (and also ependymal) lining create a special compartment in which microglia are embedded. Suited for the neuronal functions, this milieu instructs and requires also the functions of microglia. They may vary as vascular and other anatomical properties differ throughout space. Nevertheless, local variations are likely also imposed by intrinsic, namely cellular, elements of the tissue regions, as stressed below. Most essentially, microglia may after all identify themselves as being (i) distinct from extraneural tissue macrophages, (ii) and distinct from other CNS-associated myeloid cell populations as well as (iii) representing a blend with closely related phenotypes but still discrete functional capacities (Carson et al., 2007; Hanisch and Kettenmann, 2007).

## HETEROGENEITY OF MICROGLIAL RESPONSES AND RESPONDERS

While the concept of monocyte/macrophage diversity due to origin and location has a longer history (Treves, 1984; Naito et al., 1996; Sunderkotter et al., 2004), a conscious transfer to microglia could now take ground as well. More and more studies also address microglia in the aging brain, variations by species and gender or the surprising differences among individuals (Mouton et al., 2002; Meeuwsen et al., 2005; Sierra et al., 2007; Streit et al., 2008). Analyses of this kind may need to deal with a sophisticated characterization of microglial properties at close inspection and even at the single-cell level.

### CONSTITUTIVE HETEROGENEITY BY PROTEIN EXPRESSION

In contrast to the morphology, expression of proteins suited for the identification of microglia among neural cells (i.e., neurons, astrocytes, oligodendrocytes) is not equally selective when distinguishing microglia from other myeloid cells. Certain molecules (namely proteins and carbohydrates) allow the visualization of microglia in animal and human tissues as well as cell cultures although their physiological functions and sometimes even the exact nature are largely unknown. This concerns, for example, moieties bound by certain lectins. Other molecules are characterized as receptors, adhesion molecules or as an intracellular calcium-binding factor, including CD11b/CD18 (αMβ2 integrin, CR3, MAC1) or Iba1 (Kettenmann et al., 2011; Prinz et al., 2011; Greter and Merad, 2013). CD16/32/64 (FcγRIII/II/I), CD45 (leukocyte common antigen, LCA), CD68 (macrosialin), CD115 (CSF1R), CD163 (scavenger receptor, ED2), CD169 (sialoadhesin, siglec 1), CD204 (MSR), CD206 (mannose receptor), dectin-1 (β-glucan receptor), and F4/80 have been serving in the detection of microglia, although levels of expression vary (mostly increase) with or even depend on activating challenges, thus potentially biasing detection for activity states (Kettenmann et al., 2011).

Due to shared expression, most of them would not allow an unequivocal identification of microglial cells when monocytic infiltrates intermingle with them in a lesion, especially when also the microglial morphology is altered. Factors, like CD45, still discriminate microglia from perivascular and peripheral cells since expression is low(er) and can thus serve discrimination especially in flow cytometry. Other proteins are suggested to identify circulating and recently invaded monocytes/macrophages, like macrophage-related proteins (MRPs) 8 and 14 (S100A8 and S100A9), whereas again others fail to be unique to microglia or have limited value for certain species (Kettenmann et al., 2011). Accordingly, combinations of two or more markers are more practicable or even required, depending on the study and the biological material. The discriminating potential of newly identified genes with selective expression in microglia still needs confirmation, but could offer additional options (Donnou et al., 2005). Inside the CNS, expression of CX_3_CR1, the receptor of the chemokine CX_3_CL1 (fractalkine), is restricted to microglia (and other brain macrophages). In turn, CCR2 as the receptor for the monocyte-attracting chemokine CCL2 (MCP-1) is expressed by cells in the blood stream, but under non-inflammatory conditions not found within the CNS – a patho/physiological relevant pattern that has been technically extremely useful (Mildner et al., 2007; Saederup et al., 2010; Prinz et al., 2011; Tremblay et al., 2011; Mizutani et al., 2012). The availability of transgenic animals with fluorescent reporter proteins has thus tremendously fostered studies on the rate and impact of monocytic infiltration of the CNS.

Several of the above listed as well as additional molecules representing surface receptors, cytokines and trophic factors or enzymes reveal inhomogeneous expression by microglia, at least in terms of detectable presence at mRNA and protein level or signaling organization. A short list has been compiled in recent reviews and includes CD11c, CD34, CD40, CD45, CD86, CXCL14, FcγRII, IGF-I, IL-6, inducible nitric oxide synthase (iNOS), certains integrins, MHC II, neurotrophins, Tim-3, TNFα, Trem-2 as well as ion channels (Kettenmann et al., 2011; Hanisch, 2012, 2013).

One has to acknowledge that consciously presented and interpreted evidence for heterogeneity among microglia in terms of selective or preferential expressions of a molecular structure can be found in the literature records of more than a decade ago (Rezaie and Male, 1999). Even though respective glyo/protein(s) had occasionally unidentified functions such work pointed to the uniqueness of microglia in the myelomonocytic lineage and documented variations among microglia. In rat brain and spinal cord, two cell populations were identified by the expression of the 5D4 keratan sulfate (KS) epitope. Occurring as of postnatal week 21, KS associated exclusively with ramified, but not with (activated) ameboid microglia (Wilms et al., 1999). It was found to be absent from other CNS macrophages (namely in the choroid plexus, vessel walls or meninges). Also peripheral monocytes/macrophages lacked its expression. KS^+^ microglia distributed ubiquitously, but not uniformly throughout the CNS. Expressing cells were enriched in some regions and almost absent in others. These cells prefered gray over white matter as location and the global pattern suggested a favor of tissues with neurogenesis potential. The partially intimate contact to neurons pointed to a nursing function. Like for the *in situ* investigation, KS^+^ microglia comprised a subset *in vitro*, among cells expressing the CR3 or ED1 epitope without being affected by lipopolysaccharide (LPS) or interferon-γ (IFNγ) stimulations. Thus, while certain molecules (or at least epitopes) reveal a more or less pan-microglial or even general macrophage expression under basal and/or activated conditions (e.g., CR3, or in one of our studies MHC I as shown below), others can apparently discriminate microglial subpopulations by discrete anatomical distribution, white *versus* gray matter association and timed or transient appearance during ontogeny. Notably, subtypes can occur within a circumscribed region.

One of the most systematic studies thus far could determine regional expression profiles based on *ex vivo* flow cytometric analysis of CD11b, CD40, CD45, CD80, CD86, F4/80, TREM-2b, MHC II, CXCR3, CCR7, and CCR9 in isolated microglia (de Haas et al., 2008). Another study from this group related the CXCL10/CXCR3 system to the differential involvement of the local microglia in excitotoxic actions on neurons in in the mouse hippocampal regions CA1 and CA3, as triggered by *N*-methyl-D-aspartic acid (NMDA) administration (van Weering et al., 2010). This demonstration goes beyond mere detection of expression levels by identifying functional implications of a ligand-receptor signaling in microglia, by presenting a consequence for the neuronal environment as well as by showing the distinguished microglial impact for tissue regions in close vicinity. Expression and activity features would therefore not only vary between gross-anatomical structures but even within short ranges.

Going even further by the niveau of local resolution, microglial cells in side-by-side position could be shown to dissociate as to an altered protein expression – indicating a split in their individual duties. The triggering receptor expressed on myeloid cells-2 (Trem-2) is expressed by microglia under normal conditions and down-regulated when the cells are challenged. Such challenges can be achieved with LPS, a major cell wall constituent of Gram-negative bacteria signaling through TLR4, or with IFNγ, the immune IFN produced by natural killer (NK) cells and T cells of the Th1 type (Schmid et al., 2002). Both factors can drive a microglial phenotype that is well-known from studies on macrophages and has been termed as classical (also M1) or innate activation, as discussed below (Gordon and Taylor, 2005; Hanisch and Kettenmann, 2007; Mosser and Edwards, 2008). As a receptor with relevance for microglia and phagocytosis, Trem-2 is in the focus of research on autoimmune, neurodegenerative and (neuro)inflammatory diseases, including multiple sclerosis and Morbus Alzheimer (Piccio et al., 2008; Neumann and Daly, 2013). Besides other proteins, Trem-2 expression may also give hints to a heterogenous organization of microglia (Schmid et al., 2009). Not all microglia do express Trem-2 and this distinction is seen between as well as within CNS regions (Schmid et al., 2002). Interestingly, microglia in areas with incomplete BBB exhibited lowest expression. It is also interesting to speculate that plasma factors probably sensed by such a microglia are responsible for the reduced expression. Indeed, the plasma contains factors that can signal via TLR4 and that install in microglia a response that is in part similar to the reactive phenotype induced by LPS (Regen et al., 2011; Hanisch, 2013). Intriguingly, microglial cells in immediate vicinity to each other were reported to clearly differ by Trem-2 expression (Schmid et al., 2002). This is a conscious notion of intralocational distinction by protein expression – published a decade ago. In other studies, such information can likely be contained in the data without an explicit interpretation. Along this line and with conscious conclusion, microglia subtypes were recently described with regard to myelin removal under both physiological and pathophysiological conditions (van Rossum et al., 2008; Fitzner et al., 2011; Regen et al., 2011; Scheffel et al., 2012). Moreover, also TLR4- or IFNγ receptor-driven responses by cytokine release and expression of other surface molecules unraveled the existence of responder subtypes (Scheffel et al., 2012).

### HETEROGENEITY IN HOUSE-KEEPING FUNCTIONS

The never-resting microglia seem to cover also a particular function in the normal turnover of myelin (Fitzner et al., 2011). Oligodendrocytes create the extremely well-organized and also tremendously elaborate myelin structures that require constant upkeep. Apparently, they thereby rely on the assistance of microglia. Oligodendrocytes can wrap myelin material from their turnover process in exosomes and deliver them to the microglia in their surrounding. The principle of outsourcing has been recently described, both *in vitro* and *in vivo*, and involves a subpopulation of microglia for clearance (Fitzner et al., 2011). Microglia taking up these myelin-laden exosomes would not upregulate MHC II when, for example, being stimulated by IFNγ. The assumption that this group of microglia would then also not function as antigen-presenting cells got confirmed in respective T cell activation assays. The exosome uptake, that is carried out by macropinocytosis, does otherwise not trigger concomitant responses, such as release of proinflammatory mediators, nor does it lead to overt signs of “activation” within the tissue. On the other hand, challenges by inflammatory stimuli, such as IFNγ and LPS, led to a decrease in exosome internalization. In turn, those microglial cells increasing MHC II levels upon IFNγ treatment were obviously devoid of exosomal clearing activity. As a conclusion, we formulated the hypothesis of an immunologically silent disposal of endogenous material (Fitzner et al., 2011). As a service for oligodendrocytes, myelin would be constantly routed to a specialized cellular compartment, i.e., a pool of microglia, from which it could not be “accidentally” presented to the adaptive immunity. One may speculate that a disturbance in this segregation could be detrimental, and there might be actual links to the triggering of myelin-afflicting autoimmune diseases, such as multiple sclerosis. Such a daily function in the turnover of self-derived material would be distinct from and exceed phagocytotic removal of aberrant cells and structures during development (Schlegelmilch et al., 2011).

The necessity of such microglial functions during development as well as later on is further illustrated by the impact of dysfunctional microglia on higher CNS functions. In an animal model of the human obsessive-compulsive disorder spectrum, microglia with a mutation in Hoxb8 associate with behavioral symptoms of compulsive grooming and hair removal (Chen et al., 2010). Microglia with a MECP2 gene mutation and showing insufficient phagocytotic performance were characterized as a cellular substrate of pathology in a mouse model of Rett syndrome, which is known in humans as an X-linked autism spectrum disorder (Derecki et al., 2012). Paving the way for therapeutic strategies, both studies presented improvement of the respective symptoms by substitution of the defective microglia via a transfer of wildtype bone marrow and monocytic engraftment. In part, the data may also indicate that a genetic defect can deteriorate the performance of especially some yet not all microglia (Chen et al., 2010).

Life-long consequences for the CNS and the functional behavior of its microglia can also result from complications of non-genetic nature. In this regard, intrauterine and postnatal infections may not only impede proper development but leave traces in form of an increased susceptibility of the CNS to subsequent challenges later in life, involving altered responses of microglia as a causative element (Bilbo et al., 2005; Stolp et al., 2005, 2009; Bilbo and Schwarz, 2009; Monji et al., 2009), as also discussed recently (Hanisch, 2013). Yet little is known how exactly such early challenges of the immune defense actions of microglia are preserved in altered properties to be conveyed to later ontogenetic periods and whether such a priming would occur in any or preferentially in certain cells. Specialized microglia may, in addition, be required for the support of neurogenesis and oligodendrogenesis, roles that have emerged more recently (Butovsky et al., 2006b).

The principle of specialization by functions of homeostatic maintenance seems to include the in-tissue renewal of microglia itself. Tissue macrophages in general can maintain themselves (Yona et al., 2013). In the absence of a pathology or experimental stimuli the replenishment of microglia from extraneural sources, such as the bone marrow and circulating monocytes, is very low to negligible (Ajami et al., 2007, 2011; Mildner et al., 2007). Or better to say, there is no clear evidence for any replenishment during adulthood. The stability of the CNS population must thus be guaranteed by – at least some – self-renewal, which may depend on specialized cells with some “stem cell” attributes (Gomez Perdiguero et al., 2013). Microglia with such a capacity may (or even should) scatter throughout the CNS tissues, even though some regions seem to harbor populations of a higher proliferative potential than others (Marshall et al., 2008). In this regard, the subventricular zone can claim a prominent status and microglia in this niche apparently also support neurogenesis (Walton et al., 2006; Thored et al., 2009). Proliferation under hoemostatic as well as pathological conditions may thus rely on subsets rather than the entire population. Such a notion could be supported by the assumption that a cell passing through the cell cycle for division may not simultaneously mount a production of cytokines, chemokines or other proteins for executive function. If challenged by activating factors and situations that require rapid functional responses as well as an expansion of the local cell numbers the affected population of microglia may split these duties by subsets.

Increase in microglial numbers will support the capacity to fight an infection and to cope with the demands of an injured tissue. Yet inflammatory infiltrates will also contain monocytic cells, that present with functional heterogeneity and distinct roles throughout the course of a disease (Butovsky et al., 2012; London et al., 2013). They are probably needed, in addition to merely provide more cells *per se*, for some functional expertise not sufficiently covered by resident microglia. Would dividing microglia, on the other hand, create also offsprings with different properties or cells that are omnipotent at a more immature level? In some aspects, microglia may also base their functional plasticity on a kind of immaturity (Carson et al., 1998; Ladeby et al., 2005). Furthermore, microglia can migrate to a lesion site, experimentally shown by an injection of a neurotoxin or by the local puncture of a blood vessel using a laser. Areas from which they emigrated would need some subsequent repopulation to avoid a drop in microglial support activity. This could be achieved either by remigration or limited proliferation – or via monocytic engraftment (Varvel et al., 2012). Would such areas regain diversity of microglia subtypes or just be helped by a “stopgap solution”? If constant (phagocytotic) clearance of endogenous material is one of the important roles of microglia in their territorial organization, understaffed tissues could run into trouble. Phagocytic deficiency likely causes severe complications (Fitzner et al., 2011; Derecki et al., 2012). On the other hand, microglial clearance functions may have distinct and different impacts under the various disease conditions, depending on the particular pathology as well as the phagocytic cargo material, and with other cell populations proving sometimes more relevant (Mildner et al., 2011). Yet homeostatic phagocytosis is still not properly understood and would conceivably represent just one of several house-keeping functions. The importance of synaptic inspections may just be raised again as another example (Wake et al., 2009).

The use of the term “heterogeneity” in direct combination with “microglia” has thus far almost exclusively been reserved for the different responses that are triggered when exposing cells to a range of experimental stimuli and activating conditions, or when dealing with the respective diversity of reactive phenotypes in *in vivo* settings. Indeed, macrophages – and as more and more appreciated also microglia – display an elaborate spectrum of programs for adapted gene inductions and supragenetic organization of functions in order to react to diverse disturbances of the tissue homeostasis. For further reading, a number of original contributions and reviews could be suggested (Gordon and Taylor, 2005; Martinez et al., 2006, 2013; Kono and Rock, 2008; Mosser and Edwards, 2008; Michelucci et al., 2009; Ransohoff and Perry, 2009; Rivest, 2009; Pollard, 2009; Biswas and Mantovani, 2010; Piccinini and Midwood, 2010; Pukrop et al., 2010; Kettenmann et al., 2011; Olah et al., 2011; Saijo and Glass, 2011; Hanisch, 2012, 2013).

### DIVERSITY OF REACTIVE PHENOTYPES

Not so long ago, macrophages were underrated for the flexibility in their responses, regarding both the versatility of reactive programs as well as the options to further adjust or shift their individual profiles. This has changed drastically. Seminal discoveries and ground-preparing achievements being done by immunological research on (initially extraneural) macrophages resulted in refined concepts of reactive phenotype diversity (Treves, 1984; Mantovani et al., 2004; Gordon and Taylor, 2005; Martinez et al., 2006, 2009; Weber et al., 2007; Mosser and Edwards, 2008; van Rossum et al., 2008; Pollard, 2009; Biswas and Mantovani, 2010; Daley et al., 2010; Wong et al., 2010; Mikita et al., 2011; Murray and Wynn, 2011). Over the last years, this concept was rapidly implemented in the research on microglia as well, based on diverse approaches as well as CNS-adapted models and revealing both similarities in principles and profiles – but also indicating tissue- and cell type-specific response properties. The following references, covering original contributions and surveys, are selected for further reading (Popovich et al., 1999; Schmid et al., 2002; Town et al., 2005; Butovsky et al., 2006c; Colton et al., 2006; Schwartz et al., 2006; Hanisch and Kettenmann, 2007; Ponomarev et al., 2007; Davoust et al., 2008; van Rossum et al., 2008; Colton, 2009; Kigerl et al., 2009; Michelucci et al., 2009; Tierney et al., 2009; Pukrop et al., 2010; David and Kroner, 2011; Kettenmann et al., 2011; Saijo and Glass, 2011; Olah et al., 2011, 2012; Hanisch, 2012; Starossom et al., 2012; Zhou et al., 2012).

In its fundaments, the notion relies on the observation that exposure to certain cytokines, such as the T helper (Th) cell type 1 cytokine IFNγ or the pluripotent TNFα, and challenges by microbial agents, such as LPS, other TLR-agonistic compounds or pathogen-associated molecular patterns (PAMPs), would install a “classical” activation of macrophages, that has been more or less synonymously termed as a M1 phenotype. Such cells are defense-oriented, release proinflammatory and cytotoxic factors, support Th1 type of immune response – yet potentially and occasionally also at the costs of damage. Th2 cytokines, like IL-4 or IL-13, instead organize for an “alternative” activation of macrophages, also known as M2 phenotype. The respective profile is more anti-inflammatory, inflammation-resolving. It is involved in parasite killing and supports tissue repair. Differing from the uncommitted M0 condition, the M1 and M2 phenotypes demonstrate distinct sets of active genes. Their profiles are largely discrete and partially reciprocal. By certain genes, however, they can also overlap, rendering an isolated factor often rather inappropriate to reliably classify the orientation. For simplicity, the reciprocal expression of IL-10 (M2) and IL-12 (M1) served as a phenotype indication.

Classically activated macrophages were then also reported for an expression and/or release of TNFα, IL-1, IL-6, IL-23, CCL2, CCL3, CCL5, reactive oxygen and nitrogen species, matrix metalloproteinases (including MMP-1, -2, -7, -9, and -12), cyclooxygenase 2 (COX2), iNOS, the FcR types CD16/32/64, MHC II and the accessory molecules B7.1/7.2 (CD80/86), that serve in antigen presentation. Actual sets, combinations and intensities vary with the type of macrophage and stimulus. Alternatively activated macrophages show, besides the typical IL-10, expression or release of IL-1 receptor antagonist (IL-1ra), CCL17, CCL22, IL-1RII, CD163, scavenger receptors, factor XIII, fibronectin, COX1, arginase 1, and enzymes for extracellular matrix (ECM) repair (Hanisch, 2012).

The various genes and their products are not necessarily induced under every phenotype-conform condition, as mentioned above. Furthermore, the M1-*versus-*M2 classification does not reflect the versatility – and even the kind of continuum – of the observed spectra. IFNγ, for example, induces MHC II in both macrophages and microglia. LPS, driving an innate or M1-like activation, however, fails to induce it in microglia (van Rossum et al., 2008; Scheffel et al., 2012). So, not all M1 forms come with an congruent gene set, whereas certain gene products may be found in otherwise distinct phenotypes (Mantovani et al., 2004; Edwards et al., 2006). While these variations enrich the phenotype diversity even within the M1 domain (Starossom et al., 2012), different types of M2 orientations were also noted already early in these studies (Mantovani et al., 2004). Exposure to either IL-10, antibody-antigen (immune) complexes or their combinations with TLR and other agonists, contact to cellular breakdown material along with its phagocytotic removal, contact to apoptotic cells or association with a tumor install phenotypes that share M2 likeness, while simultaneously displaying sufficient differences to allow for subclassification as to M2a, M2b, and M2c. Consequently, not isolated markers but profiles define the actual state of the cells, while stages in a response process, lack of typically indicative or concomitant expression of “opposite” markers may hamper simple definitions and blur phenotype borders (Gerber and Mosser, 2001; Anderson and Mosser, 2002; Anderson et al., 2002; Town et al., 2005; Edwards et al., 2006; Mosser and Edwards, 2008; van Rossum et al., 2008; Kettenmann et al., 2011). The dynamic nature of responses cannot be emphasized enough (London et al., 2013). Monocytes/macrophages likely display distinct molecular signatures as they correlate with distinct activities and functions during the initiation, progression and resolution of a disease and the reaction to it. In addition, phenotype inductions in microglia and extraneural macrophages by standard stimuli, such as LPS, IFNγ, or IL-4, can lead to similar yet discrete profiles of induced genes and functional consequences (Butovsky et al., 2006c; van Rossum et al., 2008; Girard et al., 2013), as previously discussed (Kettenmann et al., 2011; Hanisch, 2012, 2013).

New candidate proteins and carbohydrate moieties allow now a fairly reliable association with reactive phenotypes as to their bifurcated polarity or their distinguishable varieties. To name a few, claudin (Cldn) 1, 2, and 11, found in inflammatory zone 1 (FIZZ1), mouse macrophage galactose-type C-type lectins (mMGL) 1 and 2, sphingosine kinase-1, TNF superfamily member 14 (LIGHT), transglutaminase 2, the secretory lectin chitinase 3-like protein 3 Ym1 or O- and N-glycans sort with M1 and M2 orientations, although functions in general and in regard to the phenotype are often unclear (Chang et al., 2001; Raes et al., 2002, 2005; Colton et al., 2006; Edwards et al., 2006; Maresz et al., 2008; Starossom et al., 2012; Van den Bossche et al., 2012; Zhou et al., 2012; Martinez et al., 2013).

It is important to note – also for our discussion on heterogeneity – that phenotypes are chosen at the beginning of the activation process, based on initial sets of information on threatening events. Gene expression may subsequently shift, organized by cell-autonomous mechanisms (taking late induction of IL-10 as an example), or due to altered conditions (e.g., when primary stimuli fade upon successful clearance, as for microbes) or with modulatory factors gaining influence (like cytokines, resolvins, and other mediators that are emitted by infiltrating neutrophils, monocytes, or T cell populations). Reactions may start with a defense orientation that later on deescalates and fades when profiles are required that facilitate structural repair and functional restoration. In the living tissue, phenotypes ideally induced in experimental settings as of M1- or M2-like blends could probably develop as transient stages of a complete response. We have discussed these issues in recent reviews (Kettenmann et al., 2011; Hanisch, 2012).

Notebaly, macrophages do not only commit to diverse phenotypes upon defined challenges. More or less pronounced overlaps between functionally related profiles could (and actually do) exist – especially in the more complex *in vivo* situations – as many genes, including those with an “inflammatory connotation,” are important in several responses to (as well as following to) infection and injury. Phagocytotic activity and proinflammatory components are needed for wound healing and repair. On the contrary, inflammation is a double-edged sword in cancer development and progression. It can even be supportive, and macrophages can display a bewildering variety of phenotypic markers and their combination in tumor association, as they do in many other disease situations, such as in atherosclerosis, or repair (Mantovani et al., 2004; Mantovani et al., 2009; Pukrop et al., 2010; Mantovani et al., 2013). They can exhibit phenotypic profiles that do not easily sort by a strict M1/M2 dichotomy. Also microglial populations can present with more complex locotemporal patterns of their morphological, molecular and functional features (Kigerl et al., 2009; Morrison and Filosa, 2013). Evidence for the parallel existence of M1- and M2-oriented cells in a mouse spinal cord injury model either points to hybrid states expressing genes of either activation type or suggests discrete cellular subsets following own time courses and having independently organized programs (Kigerl et al., 2009). Orientations could be split between the resident microglia on one and infiltrating monocytes on the other side – or the lines of demarcation are not that simply drawn by CNS-intrinsic *versus* extraneural origin. While it is obviously not a trivial task to assign a certain reactive phenotype as a dominat as well as persisting response pattern of macrophages and microglia to a given pathology, in some cases a major orientation and the respective molecular and cellular mechanisms can be successfully linked to a disease (Heneka et al., 2013).

### RESPONSE HETEROGENEITY UPON CHALLENGES

An increasing number of already characterized (and probably still to be identified) factors of exogenous (microbial), endogenous and especially CNS-intrinsic (homeostatic and damage- or degeneration-related) factors takes influences on the activity state and activity recruitment of microglia, as listed previously (Hanisch, 2002; Hanisch and Kettenmann, 2007; Kettenmann et al., 2011; Hanisch, 2012, 2013). They differ by origin and the situations in which they appear in microglia activation-relevant concentration and format, they belong to most disparate classes as to their chemical structure, they employ diverse modes of action and they definitely vary by their phenotype instructions.

As a principle to set an alert and to trigger a rapid transition from the surveillance (“resting”) mode to executive states, signals appear in the form of receptor agonists that are normally not found in the microglial environment – not at all, not with relevant modification or arrangement (e.g., oxidized or aggregated forms) or at effective concentration. Many classes of compounds that play roles in microglial instructions for phenotypic commitments follow this principe of “on signaling,” in which already expressed surface or intracellular receptors are stimulated by the arrival of their cognate ligands (Hanisch and Kettenmann, 2007; Kettenmann et al., 2011; Hanisch, 2012). Major examples relate to cytokines. Typically, “on signaling” occurs with the assortment of evolutionary conserved structural motifs in glyproteins, lipopeptides, glycolipids, RNA, and DNA of virus, bacteria, fungi, and protozoans, commonly described as PAMPs, which are sensed by germ line-encoded pattern recognition receptors (PRRs), notably NOD-like receptors (NLRs), RIG-I-like receptors (RLRs), C-type lectin receptors (CLRs), absence in melanoma 2-like receptors and TLRs (Kawai and Akira, 2010; Takeuchi and Akira, 2010).

In other situations, when damage or impairment has to be sensed by microglia in the absence of infection, endogenous factors can act as “activators” to drive an innate immune reaction as a sterile inflammation (Chen and Nunez, 2010; Stewart et al., 2010). Classified as damage- or danger-associated molecular patterns (DAMPs), these disparate molecules (mainly but not only proteins) usually serve most diverse functions and gain the role as danger signs only upon unphysiological release (from a cell), translocation (from the plasma to the parenchyma) and modification (e.g., oxidation; Rubartelli and Lotze, 2007; Lotze et al., 2007; Kono and Rock, 2008; Zhang and Mosser, 2008; Piccinini and Midwood, 2010). DAMP activities have been established for (to name a few) chromatin-associated and other intracellular proteins, such as high mobilioty group box protein (HMGB) 1, S100A8, and S100A9, a range of heat shock proteins, ECM and plasma components, such as tenascin, versican, fibronectin, lactoferrin, lipoprotein A, and oxidized low density lipoprotein, but also serum amyloid and amyloid β (Aβ; Hanisch, 2012). New entries to the list are reported on a constant basis, recently regarding fetuin (Pal et al., 2012). Interestingly, many of these DAMPs are recognized by the binding to PAMP receptors, most notably TLRs. By the recognition of alarming structural patterns a rather small number of such receptors can cover a wide range of pathogens as well as indicators of dangerous situations – and the reactions to these threats would be immediately in the hands of the sentinel cells, like microglia in the CNS (Hanisch, 2012, 2013).

In contrast to this “on signaling,” microglia can, in turn, also be alerted and “activated” when certain calming influences fade or disappear. Accordingly, such an organization would be a kind of “off signaling,” the term being introduced as a microglial principle already in 2004 (van Rossum and Hanisch, 2004; Biber et al., 2007; Hanisch and Kettenmann, 2007; Hanisch, 2012). For first pairs of ligands and their receptors, reciprocal expression by neurons and microglia has been shown to have a controlling influence, such as in the cases of CD200/CD200R, CD47-SIRPα, CX_3_CL1/CX_3_CR1 and CD22/CD45. Yet microglia may sense the decline of neighboring neurons also via altered neurotransmitter levels. Both sources of information could be important for microglia to rapidly respond to any homeostatic disturbance affecting neurons simply on the basis of lost inputs, and without the need of expressing a sweeping array of particular receptors (Hanisch, 2013). In other words, danger is recognized, also by microglia, via a whole tool box of receptors and signaling systems (Lotze et al., 2007; Matzinger, 2007; Rubartelli and Lotze, 2007; Lehnardt et al., 2008; Kettenmann et al., 2011). In harsh contrast to the growing knowledge on signals and signaling principles that keep the control over microglia as a major cell type in the CNS, much less information is yet delivered as to the individuality of the cellular responses.

Injections of LPS into rat neocortex, hippocampus or the substantia nigra revealed a markedly distinct susceptibility in terms of neurodegeneration, with a profound impact on mesencephalic and especially dopaminergic neurons (Kim et al., 2000). The critical role of microglia was addressed in respective neuron-glia culture preparations. Sensitivity of mesencephalic and resistance of cortical and hippocampal neurons both *in vivo* as well as *in vitro* apparently matched the region-selective microglial responsiveness and responses. The substantia nigra is the CNS region in rodents that accommodates the highest density of microglia (Lawson et al., 1990). Respective cell transfer (supplementation) experiments could associate cell number and outcome. Yet quantity might not explain all regional differences. Neonatal rat microglia isolated form brainstem, cortex, hippocampus, striatum, and thalmus differently responded to glutamate, ATP and LPS (Lai et al., 2011). This report and other work with functional demonstrations (van Weering et al., 2010) correlate to the inventories of distinct expression profiles (de Haas et al., 2008) and precipitate the conclusion as to region-specific microglial populations. Of course, the ontogenetic stage of the microglial sample cannot be neglected since several features, especially the organization of receptor signaling and stimulation consequences, obviously mature not only prior but also after birth (Draheim et al., 1999; Scheffel et al., 2012). Nevertheless, regional distinction with varying resolution is on the way to depict responder heterogeneity in rather circumscribed areas.

In a recent study, we addressed the postnatal reorganization of microglial responses to TLR activation (Scheffel et al., 2012). Along the characterization of inducible genes and profiles of synthesized factors we also observed responder subsets, i.e., cells being responsible for the release of certain cytokines and chemokines. The respective distinction of producers from the non-producing subpopulations developed as part of a more general maturation of microglial features. This concerned profiles of genes and activities expressed under basal conditions or subsequent to a defined stimulation. Microglia were harvested from mice at birth or at the postnatal days P21 and P49 and kept *ex vivo* for various periods of time. Interestingly, and against the common expectation, several features that were studied in repeated sessions did not reveal obvious changes with the time in culture but differed, partially in a drastic fashion, with the time point during postnatal development, namely the day at which the microglia were isolated. Even though such an *ex vivo* approach would never claim to properly reflect tissue-encoded features of the cells in their entire complexity, at least some of the critical aspects seemed to be carried over to the culture where they were maintained with surprising stability.

When microglia was challenged with defined structural varianst of LPS they produced, among other cytokines and chemokines, TNFα (Regen et al., 2011; Scheffel et al., 2012). Microglia could even discriminate among the LPS chemotypes as to the actual release profiles, and this ability developed postnatally. Notably, synthesis of TNFα related to a subpopulation which increased from about 30% in neonatal to 75% in the young adult cells. At the same time, the boundaries defining the responder *versus* non-responder subpopulations – as revealed in flow cytometry – gained precision (i.e., by increased peak distance). Moreover, the two subtypes were then also seen in isolates of microglia from different regions of the adult CNS (Scheffel et al., 2012). Their existence was confirmed by immunocytochemistry on both cells and organotypic brain slice preparations *in vitro* (using also confocal microscopy) and on tissue sections from mice that underwent targeted focal experimental autoimmune encephalomyelitis (EAE).

Selective induction of TNFα was accompanied by a subset-restricted synthesis of CCL3, an important chemokine also known as macrophage inflammatory protein 1α (MIP-1α), that serves in the attraction of monocytes, T and B cells, eosinophils as well as DCs (Scheffel et al., 2012). The rather small subpopulation would apparently suffice an impressive release, since CCL3 can be induced in high amounts. Most of the CCL3-synthesizing cells were contained in the (larger) population of TNFα producers, as revealed by combined detection. This TNFα^+^CCL3^+^ subpopulation increased in size as the postnatal development of microglia features proceeded – ascertained by expression and cell cycle analyses as well as electrophysiological recordings (Scheffel et al., 2012; Hanisch, 2013).

In support of these observations, a similar subset-selective TNFα induction by LPS was also reported for rat microglia (Kawahara et al., 2009; Shen et al., 2009), ruling out a species phenomenon. Employing FluoroSpot technology for single cell analyses among populations, a comprehensive characterization of TLR-activated cytokine secretion recently revealed distinct responder subtypes among human monocytes (Smedman et al., 2011). Production of TNFα was determined in subpopulations of TLR2- and TLR4-stimulated cells. Moreover, also other cytokines and chemokines depended for their release on subsets. Interestingly, combinatory detections identified cells with co-secretion capacity for pairs of factors, such as TNFα and MIP-1β (CCL4), whereas fractions of cells in the same population revealed only singular synthesis. Moreover, the release of certain cytokines relied on surprisingly small numbers of cells. IL-12p40 production, for example, was confined to less than 10% of the monocytes. Findings of this study – focusing on human monocytes – are thus very similar to those obtained with rodent microglia.

While a developmental reorganization of the microglial TLR systems is also in line with the data by others (Kaul et al., 2012), our findings point to a rearrangement in the associated signaling cascades, rather than a change in receptor expression. Evidence indicates an altered organization of the pathway requiring the signaling adaptor myeloid differentiation primary response gene 88 (MyD88), which participates in intracellular consequences of almost all TLRs (except for TLR3). On the contrary, the adaptor protein TIR-domain containing adaptor protein inducing IFNβ (TRIF), that conveys information under TLR3 and also for TLR4, seemd to be spared. Noteworthy, we found critical TLR4 functions to be distinctly governed by MyD88 and TRIF (Regen et al., 2011) and to undergo transient reorganization during postnatal development (Scheffel et al., 2012). Exemplified for the reactions to TLR1/2, TLR4, and TLR6/2, microglia rebuild their mechanisms during a critical window within the first few weeks after birth (marked by a peak around p21) – while simultaneously passing through a gradual change of other properties. Microglia will acquire the typical morphology of the “resting” cell (which is not dormant, as we know), accompanying the maturation of the CNS in terms of its neuronal circuitry and myelination (Scheffel et al., 2012). As this period coincides with a suggested phase of increased susceptibility to CNS infection, we also tested mice for the outcome of an intracerebral bacteria deposition and noticed a higher mortality for those at P21, in comparison to animals at an age of P49.

The formation of TNFα- and CCL3-producing subsets of microglia under LPS was not related to a lack of TLR(4) in the complementary cell fraction. When studying microglial inductions upon TLR4 challenge, we found a strong expression of MHC I in almost all cells. While failing to induce MHC II under these conditions, LPS caused a panpopulational response in microglia at any age and in all regions examined (Scheffel et al., 2012). MHC I^+^TNFα^-^ and MHC I^+^TNFα^+^ microglia thus do not split by their TLR4 expression and responsiveness as such. Among the TLRs, TLR4 appears to be homogeneously distributed over macrophage populations (Gautier et al., 2012). In the case of microglia, cells must rather differ by TLR4 signaling. Under the influence of TLR4-agonistic PAMPs or DAMPs, subsets could provide individual elements to compose the reactive phenotype of a responding microglia population. Some deliver, for example, TNFα to which other cells have then to obey, while all of them identify themselves with MHC I expression. Since microglia differ by their TNFα receptor expression (Kraft et al., 2009), this could create a hierarchy of cells, with some in control of the synthesis and others with a varying level of perception. Cells with preferential expression of enzymes, such as COX2 or iNOS, could similarly serve as (privileged) sources of prostaglandines and NO (Kawahara et al., 2009; Scheffel et al., 2012). Reactive phenotypes may thus reflect an ensemble of individual cellular engagements. Inhomogeneous responses of microglia in circumscribed populations (i.e., of a defined tissue region) to a homogeneously presented stimulus could be taken as a hint to their differential organization. Distinct response capacities could be established even before the challenge. Heterogeneity could be, at least in some part, a constitutive one.

In contrast to MHC I, microglial expression of MHC II for professional antigen presentation was not triggered by TLR4 activation, but readily induced by IFNγ, both *in vitro* as well as *in vivo, *and then in a subset (Fitzner et al., 2011; Scheffel et al., 2012). Also upon injection of IFNγ *in vivo*, microglia reveal MHC II immunoreactivity (Fitzner et al., 2011). B7.1 and B7.2 (CD80 and CD86), costimulatory molecules for MHC II function and interaction with CD4^+^ T cells, also revealed induction by subsets. IFNγ injection in animals caused a scattered MHC II^+^ induction, a pattern ruling out that it just followed a gradient (Fitzner et al., 2011; Scheffel et al., 2012). Moreover, evidence for restricted expression can also be derived from other reports (Ponomarev et al., 2005, 2006; Starossom et al., 2012). We presented data from a histopathological examination of human tissue that expression of respective HLA structures in hypoxic lesions is confined to individual microglia (Scheffel et al., 2012). TLR4 signaling organization by subsets could probably relate to the organization of subsets by MHC II expression. A recent study identified a previously unknown importance of the intracellular MHC II pool in acting as an adaptor in TLR signaling (Liu et al., 2011). Conceivably, MHC II, as it is also inducible by IFNγ, would then participate in the TLR reactions of some cells, whereas TLR4 itself is functionally expressed by the majority of microglia. The well-known phenomenon of IFNγ priming as affecting microglial challenges by LPS (Häusler et al., 2002) may consequently involve unequal mechanisms in cellular subsets (Scheffel et al., 2012).

While phagocytotic and other clearance mechanisms are essential elements of the microglial activity spectrum to support CNS development as well as maintenance, removal of foreign and endogenous material is most critical in infections, injuries, autoimmune and degenerative processes (Hanisch and Kettenmann, 2007; Kettenmann et al., 2011). Successful clearance of bacteria decides already in the initial phase of an infection on the extent of damage and sequelae, while efficient removal of myelin debris is a known prerequisite for attempts of remyelination in multiple sclerosis. In other disease, deficiency in the clearance of protein deposits, such as Aβ plaques in Alzheimer’s disease, may have harmful outcomes, with the role of microglia being different in comparison to extraneural monocytes. Mechanisms of a functional – and dysfunctional – clearance are of an imaginable huge clinical importance (see also above). On the other hand, these functions have been addresses mainly for *the* microglia, with thus far little conscious distinction of potential subtypes.

Myelin uptake activity in pathophysiological conditions apparently associates with a fraction of microglia (van Rossum et al., 2008; Regen et al., 2011; Scheffel et al., 2012), similarly as it was described for the physiological disposal of exosomally encapsulated myelin (Fitzner et al., 2011). Even though the underlying mechanisms and the responsible molecular machinery of incorporation will differ between clearances of the exosomes *versus* free myelin, both are suppressed by inflammatory conditions (van Rossum et al., 2008; Fitzner et al., 2011; Regen et al., 2011; Scheffel et al., 2012). Interestingly, TLR activity regulates the myelin clearance with MyD88 signaling dependence and with opposite outcome in comparison to the phagocytosis and killing of Gram-negative as well as -positive bacterial strains (Ribes et al., 2009, 2010a,b, Ribes et al., 2011). The latter functions might also be reserved for subtypes, as work in progress suggests. Probably, this qualifies only partially overlapping sets of microglia as specialists that are either called up for duty when the CNS is under an infectious attack or take the part of clearing the terrain from debris.

The collecton of evidence for microglial responder heterogeneity could be continued as the literature can serve as a source for data and images that contain such information, although explicit mentioning is not always found in the text. Of course, gene expression studies reveal signatures that apply to microglia as a CNS-resident entity. Microglia show low expression of many transcripts which are readily found in extraneural macrophages, whereas they present with a particular profile of other genes, such as those encoding for molecules of the oxidative metabolism (Gautier et al., 2012). While such classes will be used to understand the gross-anatomical organization of myeloid cell distribution and function, it will be the identification of also minute differences that could provide an image of microglial response diversity and its probably underlying constitutive heterogeneity at high resolution. There is a list of molecules and activities, responses as well as susceptibilities with particular expression and distribution patterns, which would deserve consideration for subset analyses (Butovsky et al., 2006a; He et al., 2006; Sriram et al., 2006; Ensinger et al., 2010; Iwama et al., 2011). Some molecules, such as galectin-3/Mac-2, could thereby prove as suitable candidates for delineating sets of cells by expression and associated function (Venkatesan et al., 2010).

## INSTRUCTION AND ORGANIZATION OF HETEROGENEITY

Since the concept of microglial heterogeneity is just emerging, potential principles of its organization are still enigmatic. Several sources of instruction can, however, be envisaged. One may define orientations of cells during development. Alternatively, cells may acquire their individuality upon arrival at their tissue destination and within the respective local cell community. Still another model could be based on probability.

### HETEROGENEITY BY LINEAGE ORIGIN AND MICROENVIRONMENT

Substantial efforts based on genetic strategies and fate mapping have led to the identification of progenitors and lineages that give rise to the various myeloid cell types and thereby covering macrophages of various tissues, including microglia. Against the common believe of a mandatory monocytic replenishment, this work can strengthen concepts claiming primitive progenitors from the yolk sac as a source. It shows that major tissue macrophage populations are established before birth and kept independent of monocytes from the blood (Naito et al., 1996; Yona et al., 2013). Transcription factors have been nominated to be distinctly involved in the development of DCs, monocytes, tissue macrophages and microglia, such as Batf3, Flt3, PU.1, or Myb (Ginhoux et al., 2010; Gautier et al., 2012; Schulz et al., 2012; Satpathy et al., 2012; Gomez Perdiguero et al., 2013). Work published now adds factors and steps for microgliogenesis, confirming PU.1 and introducing Irf8 (Kierdorf et al., 2013). Accordingly, microglia derive from primitive c-Kit-positive erythromyeloid precursors in the yolk sac of the mouse that develope through stages with distinct expression patterns of CD31, CD45, c-Kit, CX_3_CR1, F4/80 as well as MCSFR and that depend for proper development and settlement also on MMP-8 and MMP-9 (Kierdorf et al., 2013). This fascinating research will continue to fill gaps in precursor sequences and help to draw road maps of tissue population (colonization). Conceivably, some late differentiation steps include further splitting of the microglial lineage to create diversity by subsets. Earlier work suggested, based on a gene defect and respective lineage labeling, that not the entire microglia population was affected, leaving room for speculation as to its heterogeneity (Chen et al., 2010). If microglial lineage branching during development would give rise to subtypes their distribution throughout the CNS would need to be outlined as well – especially to explain their settlement in overlapping territories.

Alternatively to a predisposition of cells as to their origin, tissue adaptation of molecular and functional properties could derive from local instructions and the requirements of particular micromilieus. The anatomical divisions of the CNS come with variable composition as it regards their cellular and molecular constituents. Simply sorted by white and gray matter, the regions differ by the content of myelin, a complex and vulnerable structure that by itself may determine microglial properties or set directions for the flow of messengers. Expression of receptors does vary by region, and inspection of data published earlier may indicate trends by white matter content (de Haas et al., 2008). These differences could impact on development, normal functionality or the vulnerability of CNS structures to inflammation (McKay et al., 2007; Hristova et al., 2010). Microglia from white and gray matter differ by expression of Tim-3, an immunoregulatory receptor (Anderson et al., 2007). The authors linked its differential and timed expression by microglia, DC-like as well as Th1 cells to a regulation of inflammation and immune responses in defined phases. Interestingly, heterogeneity of microglia might be complemented by heterogeneity of oligodendroglial subpopulations in subregions of gray and white matter (Kitada and Rowitch, 2006). The biochemical milieu is also determined by the vascular and ECM features. Exposure to some plasma factors at certain sites – and not only upon a BBB disruption – may affect neighboring microglia, as reflected by the distinct sialoadhesion expression (Perry et al., 1992). Microglia are not just embedded in an ECM environment for structural support. Interactions are not limited to the manipulation and rebuilding of ECM by microglial proteases, such as upon an injury or cell migration, but include signals to microglia, for example via integrins. Certain factors are – in varied format – present in both the plasma and the ECM, like fibronectin, that can potentially trigger microglial responses through TLR4 (Regen et al., 2011).

Structures of the CNS vary by prevalent neurotransmitters, for example in the cerebral cortex by the layers. Microglia express a range of classical neurotransmitter and purinergic receptors and also respond to their activation (Pocock and Kettenmann, 2007). Accordingly, they may sense acetylcholine, dopamine, glutamate, noradrenaline, GABA, and purines, namely ATP and metabolites (Kettenmann et al., 2011). In addition, the repertoire of receptors covers those for neurohormones, neuromodulators, steroids, prostaglandins, leukotrienes, complement, and immunoglobulins in order to respond by various outcomes to opiodis, angiotensin, bradykinin, endothelin, neurokinins, neurotrophins, somatostatin, vasoactive intestinal polypeptide, cannabinoids, histamine, platelet activating factor, glucocorticoids, and mineralocorticoids (Kettenmann et al., 2011). Microglia have been shown to express receptors for proteases, such as thrombin, which may come into play when an injury causes inundation of the parenchyma by plasma content but also when such factors are operating within the intact CNS (Balcaitis et al., 2003; Hanisch et al., 2004). Their importance may differ and vary with the ontogenic stage or homoeostatic situation. Some may serve as modulators to synergize with or to contain the consequences of cytokines, chemokines or microbial agents (Kettenmann et al., 2011). Undoubted is, however, that they would exert control on different microglial cell populations differently depending on the local levels of their agonists. Distinguishable expression profiles of such receptors would determine distinct functional adjustments. Yet even more classical neurotransmitter receptors may have some not yet fully appreciated implications in a general, phasic or disease-related control of microglia and with specificity by receptor properties and outcomes by region (Zerrate et al., 2007; Heneka et al., 2010). For CNS tissue structures and their microglia, neurochemical cues could be critical determinants. However, would tiny differences or flat gradients of instructing factors be sufficient to govern differential subtype orientation for cells in close vicinity?

### RESPONDER SUBTYPES BY STOCHASTIC VARIATION

The above mechanisms would define individual capacities of microglia by some constitutive principle. Cells would be pre-determined by lineage commitment or by their environment. Yet variety in responses, such as profiles of induced genes, could also be explained by probability (Hume, 2000). This exciting concept describes transcriptional regulation in individual cells by probabilistic events and could explain heterogeneous expression among a cell population as occurring by chance. The concept explicitly applies to leukocyte differentiation and activities. It thereby even offers tempting options for explaining the observation of subpopulations. Key to the model is the variable stability of the respective mRNAs and their translation products, i.e., proteins. A physiological “purpose” of such a simply stochastic organization could derive from the generation of an entire spectrum of individual responses in individual responders. Such a repertoire could then cope with any challenge. Indeed, heterogeneity in the responses of a macrophage cell line to LPS exposure was subsequently shown to arise from autonomous probability in the transcription of individual genes, including TNFα (Ravasi et al., 2002).

A “hatched” expression of genes could be an argument against the existence of “real,” namely pre-determined subtypes. In subpopulation analyses of any kind, some quantitative variation could pretend qualitative distinctions simply due to insufficient detection sensitivity or a variation by chance. The former situation would be a technical pitfall, the latter would stand for a basic phenomenon. A probabilistic organization of response diversity would thus not require any pre-instruction and leave the decision on subtypes to the moment of a challenge. It remains to be shown whether this is enough to explain all facets of functional diversity of microglia (and other myeloid cell populations), including house-keeping activities and activation in emergency settings. Indeed, transcriptional regulation in macrophages presents with some previously unforeseen complexity (Lawrence and Natoli, 2011). Multiplicity of approaches will avoid that conclusions are rashly drawn from single lines of evidence. In this regard, populational context may determine single-cell heterogeneity in a combination of intrinsic and extrinsic factors. Modeling approaches aim at explaining the phenomenon and to predict differences among a cell population with regard to gene transcription, phosphorylation events, cell morphology as well as drug perturbations (Snijder et al., 2009). Stochastic fluctuations in transcription factors at critical decision-making phases could participate in creating and determining (also local) diversity of microglia. Most convincing support for an existence of distinct microglia could be taken from a demonstration of the vital importance of a particular subtype and by dissecting the mechanisms by which it is instructed.

### ADJUSTMENT OF RESPONSE CAPACITIES BY EXPERIENCE

All this has to consider that microglia are a rather long-lived population that is probably maintained with some in-tissue renewal but – most likely – without replenishment from bone marrow sources, at least under healthy adult conditions (Mildner et al., 2007; Ajami et al., 2011; Gomez Perdiguero et al., 2013). If instructed for a set of (potential) functions by local cues, the setting might be either constantly maintained, similarly driven by receptor signaling as it was discussed for control of the activity level by “on” and “off” signals, or it might be induced once and preserved. Could a microglia subpopulation, for example, be experimentally translocated from its natural location to another tissue region and thereby change prominent features? It would also be important to clarify whether commitments stay stable throughout the life span of the individual cell and/or whether they may change – especially also following to a challenge. Would, for example, a transient activation episod simply lead to a return of microglia to their naïve pre-activation status or leave an engram affecting functional behavior in the post-activation phase? Would this lead to a (locally) distinct microglia?

This question has been raised but awaits further answers from experimental demonstrations of either preserved or altered microglial properties subsequent to activation (Hanisch and Kettenmann, 2007; Saijo and Glass, 2011; Hanisch, 2012). Phenomena of “priming” and “tolerance,” however, are already described. Also microglia change responses to a stimulus after being exposed to the same or another stimulus before (Häusler et al., 2002). If lasting for a longer period of time, such an altered phenotype setting could be termed “experience” or even “memory.” Traces of former gene activation remain in form of epigenetic modifications and thereby affect transcriptional activity for a long time and even through a cell cycle (Probst et al., 2009; Suderman et al., 2012). Covalent modifications of chromatin proteins or the DNA itself are major epigenetic principles. In contrast to cells of the adaptive immunity (such as T lymphocytes), which reveal distinct epigenetic changes during their differentiation (Wei et al., 2009), epigenetic regulation in innate immune cells is still largely unknown. As macrophages maintain a high degree of plasticity to commit to diverse reactive phenotypes, epigenetic modifications could be expected to take a part therein. Indeed, regulations of this kind seem to play fundamental roles in inflammatory processes and the functional control of innate immunity (Foster et al., 2007; Foster and Medzhitov, 2009).

## CONCLUSION

After all, the plural term “microglia” may include a true meaning as to the existence of subsets with preferential or exclusive duties. Subsets may specialize for day-to-day functions as well as harbor selective capacities that become effective on demand. Whether such an individuality is predestined before or organized upon arrival of microglia at their locations in the tissue – or whether it occurs upon challenge as an entirely stochastic phenomenon – still remains to be shown. Whether and how a specialization can be found similarly throughout the CNS is not known either. The concept of functional diversity is worth more consideration not for the sake of an inventory of modifications or a sorting of cells and cell activities of every description. Practical value derives from a refined classification of activities and activity states in lesions or during the course of a neuropathology. Most importantly, individual microglial subsets and (or) their functions could be addressed by targeted manipulation in experimental settings and therapeutic interventions.

## Conflict of Interest Statement

The authors declare that the research was conducted in the absence of any commercial or financial relationships that could be construed as a potential conflict of interest.
